# Leveraging femtosecond laser machining for the fabrication of tubular-based Organ-on-Chip systems: modeling cancer metastasis from invasion to intravasation

**DOI:** 10.1016/j.mtbio.2025.101926

**Published:** 2025-05-29

**Authors:** Mohammad Jouybar, Oscar Stassen, Hamed Moradi, Pan Zuo, Jaap M.J. den Toonder

**Affiliations:** a Microsystems, Department of Mechanical Engineering, Eindhoven University of Technology, Eindhoven, the Netherlands; b Institute for Complex Molecular Systems, Eindhoven, the Netherlands; c Department of Biomedical Engineering, Eindhoven University of Technology, Eindhoven, the Netherlands

**Keywords:** Cancer-on-Chip, Tubular channel, Microfluidics, Tumor microenvironment, Cancer invasion, Cancer intravasation, Metastasis, Organ-on-Chip

## Abstract

Organ-on-Chip (OoC) models often include microchannel-based vessels and ducts with rectangular cross-sections, and therefore these lack the geometry and morphology found in tubular structures in vivo. Channels with round cross-sections can better mimic the physiology and cellular behavior of tubular structures, such as (micro)vessels and breast ducts, by providing a more *in vivo*-like geometry. Here, we utilize femtosecond laser machining to integrate tubular channels in an Organ-on-Chip device; our "Lumina-Chip" contains two tubular channels, both connected to a central channel along their entire length. This versatile fabrication technique, combined with replica molding, enables us to obtain a medium-throughput version of the device, including nine Lumina-Chips. In this study, we showcase the Lumina-Chip's capability by modeling breast cancer invasion, migration, and intravasation, all within a single device as a representative application. We use the device to observe the progression of breast cancer cells from a breast duct (formed in the first lumen, lined with normal epithelial cells), through an extracellular matrix (comprised of collagen I in the central channel), and ultimately into a vessel (formed in the second lumen, lined with endothelial cells). A permeability analysis confirms that the vessel wall maintains strong barrier functionality in the absence of cancer cells. Two types of breast cancer tumoroids (invasive and non-invasive) introduced into the breast duct exhibit distinctly different invasive behaviors.

While we present breast cancer metastasis as a showcase application, the Lumina-Chip also holds potential for other biological applications where epithelial ducts and vessels with tubular structures are critical components.

## Graphical abstract

**Figure:**
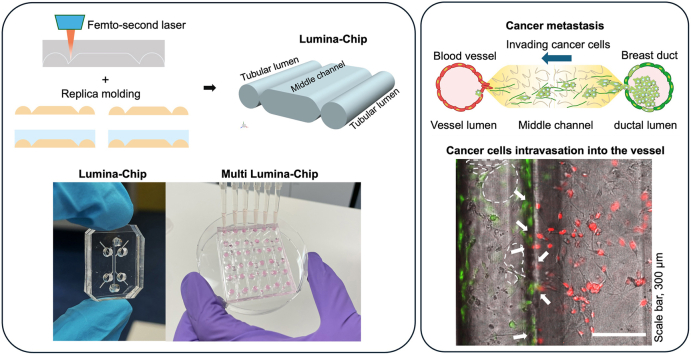

## 1 Introduction

Organ-on-Chip (OoC) models are microfluidic devices that contain microchannels and compartments, which are utilized to reproducibly create advanced 3-dimensional (3D) cell cultures within a microenvironment that is representative of in vivo conditions in the human body. The compartmentalized microchannels in these models allow for control of the cellular and extracellular matrix (ECM) compositions, fluid transport, local biochemical gradients, and mechanical cues, which together enable the emulation of the physiology, functions, and diseased states of human organs [1,2]. Most OoC models still use channels with a rectangular cross-section, which are not the best representation of the geometry and morphology of in vivo tubular structures with round cross-sections such as blood vessels and mammary ducts. The channel geometry is an important characteristic in OoC devices that influences cellular morphology [3]. It has been shown that substrate curvature and channel geometry can influence the morphology of cells cultured on them [[3], [4], [5], [6], [7]]. Hence, microchannels with circular cross-sections better mimic the physiology and cellular behavior of tubular structures such as (micro)vessels and epithelial ducts than conventional microchannels with a rectangular cross-section, by providing a more *in vivo*-like geometry and a corresponding uniform wall shear stress under physiological flow conditions [[8], [9], [10]]. We recently studied the influence of channel geometry and flow on the orientation and morphology of endothelial cells cultured in them, systematically comparing the effects of circular and rectangular cross-sections while applying different flow regimes. This study revealed that channel curvature determines cell orientation in static conditions and that adding flow results in a competing effect between geometry and flow, for which the resulting cell alignment depends on the flow conditions. These results underline the importance of mimicking physiological geometries in OoC models [3].

Recently, several techniques have been introduced to improve geometrical features in OoC systems by integrating tubular cross-section channels [[11], [12], [13]]. Early techniques include photoresist reflow [[14], [15], [16]], viscous finger patterning [17,18] and template-casting [13,[19], [20], [21], [22]]. In the resist reflow technique, the initial photoresist structures turn into rounded structures (i.e., having curved sidewalls) with typical dimensions of hundreds of micrometers and larger, by heating the photoresist beyond its softening point [14,15]. This technique was used to generate rounded molds for obtaining tubular channels forming blood vessels [16]. Viscous finger patterning relies on inducing a controlled flow of a low-viscosity liquid through a more viscous liquid, such as an uncrosslinked hydrogel, to create a hollow tubular structure [17,18]. De Graaf et al. utilized this technique to generate microvessels with an average diameter of 336 ± 15 μm within a collagen I scaffold [18]. Template-casting involves using sacrificial molds to create tubular channels also with smaller diameters (with an average size of ∼160 μm) [13,20]. Various types of templates have been utilized, ranging from rods and needles that are physically removed after curing of hydrogels cast around them, to 3D-printed structures that are chemically dissolved. More complex templates can be created by using advanced 3D-printing techniques to print sacrificial fiber networks that are subsequently cast in hydrogel or uncured polymer and finally removed using selective dissolution [[23], [24], [25]]. Recently, laser-based techniques, such as nano- and femtosecond laser (FSL) ablation of hydrogels, have been utilized as highly precise methods for the integration of curved structures in OoC models [[26], [27], [28]].

OoC models hold substantial promise as disease models for mechanistic understanding of the development and progression of diseases, and ultimately for drug development and personalized medicine [29]. Cancer-on-Chip (CoC) models, a subtype of OoC systems, have been useful for gaining an understanding of cancer metastasis, including cancer cell invasion, migration [30,31], intravasation [32], extravasation [33], and interactions with the microvasculature [22,[34], [35], [36]].

Recently, studies on the early phases of metastasis, specifically invasion from the epithelium and migration into the stromal ECM, have provided valuable insights [[37], [38], [39]]. However, CoC models that include cancer cell invasion from an epithelium, as well as subsequent critical steps such as interaction with blood vessels and intravasation into the vasculature, are still lacking.

Here, we present a new approach utilizing FSL machining as a versatile fabrication technique with a very large design freedom, allowing complex shapes with a wide range of dimensions (several microns to millimeters) on large surface areas. This technique enables the creation of CoC models with multiple connected tubular lumens. As a case study, we apply this technique to develop an advanced CoC device, replacing conventional rectangular channels with tubular channels. Using FSL machining, we produce precise molds in silica glass for the fabrication of the final device in polydimethylsiloxane (PDMS) via replica molding. Our device, called "Lumina-Chip," features two channels with tubular cross-sections connected along their entire length to a common middle compartment. Multiple Lumina-Chips can be successfully integrated into a medium-throughput multi-chip format, enabling the simultaneous testing of nine chips on a single platform, which is compatible with multi-channel pipetting and high-throughput imaging systems. As a showcase study, we apply the Lumina-Chip to investigate cancer invasion originating from a breast duct, subsequent migration, and intravasation, all within the same chip. While many studies have focused on the invasion of cancer cells into the matrix and others on intravasation or extravasation of cancer cells in interaction with blood vessels, our model allows us to emulate the complete process of the initial phases of breast cancer metastasis, including intravasation. We observe cancer cell invasion from epithelial protrusions, a step often overlooked in previous studies, followed by migration towards and intravasation into the vessel. While we present breast cancer metastasis as a showcase study, our tubular lumen-based microfluidic device can also be used for other OoC applications involving tubular structures, such as lung and kidney ducts, and their interaction with microvessels present throughout the human body.

## 2 Results

### 2.1 Femtosecond laser machining facilitates the realization of microfluidic chips with tubular luminal structures

We used femtosecond laser (FSL) machining to fabricate OoC devices containing multiple connected channels with a tubular cross-section. The focused FSL beam energy induces highly localized internal modifications of fused silica glass, resulting in an increase in the chemical etching rate by orders of magnitude. Hence, patterns written by the FSL beam are selectively removed by subsequent wet chemical etching in potassium hydroxide (KOH), enabling the realization of well-defined curved structures (Fig. 1). We used FSL machining to fabricate the master mold for the Lumina-Chip. Subsequent PDMS casting and two-step replica molding enable the high-volume production of the master-mold replicas in PDMS (Fig. 1A–F) [40]. The final device was obtained by aligned bonding of two layers, as shown in the schematic in Fig. 1G and in Fig. 1H.

**Fig. 1:**
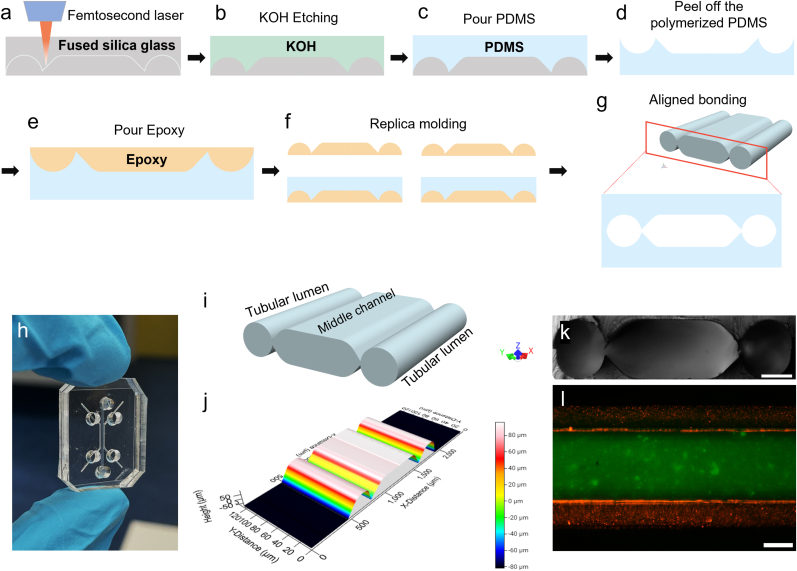
**The fabrication method of the Lumina-Chip, and its design characteristics. a**-**g**, The fabrication protocol includes (**a**) FSL machining in fused silica glass, (**b**) wet potassium hydroxide (KOH) etching, (**c**) PDMS molding, (**d**) peeling off, (**e**, **f**) two-step replica molding, and (**g**) aligned bonding. **h**, A picture of the Lumina-Chip, displaying the inlets and outlets for the two lateral lumens and the middle compartment. **i**, Schematic representation of the 3D configuration of the channels in the Lumina-Chip. **j**, The height contour of the silica glass master-mold of the Lumina-Chip; this is the profile of one half of the master-mold. **k**, Cross-section of the Lumina-Chip; the interfaces between the lateral lumens and the central middle hydrogel channel both consist of a gap or slit of 30 μm that is fully open along the entire length of the channels; this configuration was found to be suitable for keeping hydrogel in the middle channel by providing sufficient surface tension, while only minimally affecting the luminal channel geometry; scale bar, 200 μm **l**, Top view of the Lumina-Chip with collagen I (green) filled in the middle channel, and red fluorescent particles infused in the lateral lumens; scale bar, 300 μm.

The Lumina-Chip is composed of three microfluidic channels, including two lateral channels with tubular cross-sections, which are both connected to the middle channel (Fig. 1I–K). The interfaces between the two lateral lumens and the central hydrogel chamber both consist of a gap or slit of 30 μm, fully open along the entire length of the channels. This configuration was found to be suitable for keeping hydrogel in the middle channel by providing sufficient surface tension while only minimally affecting the tubular geometry of the lumens. The dimensions of the master mold for the half-device were measured using a structural profilometer, revealing a height of 150 μm (Fig. 1J). The lateral channels have a diameter of 300 μm, and the distance between the two lateral channels is 800 μm. Fig. 1l shows an image of a Lumina-Chip after infusion of collagen I in the middle channel and subsequent polymerization (stained in green), followed by infusion of the two lateral channels with red fluorescent tracer particles. The collagen I gel did not leak into the lateral channels, and the fluorescent particles did not flow into the gel, confirming the appropriate design of the interface between the lumens and the central hydrogel.

We further utilized FSL machining to fabricate a multi-chip plate to be used as a medium-throughput model that complies with well plate and Petri dish standards. The procedural methodology for the fabrication of the multi-chip plate closely paralleled that of the single Lumina-Chip (Fig. 2A). The design involved nine individual Lumina-Chips positioned adjacent to each other, configured in a 3 × 3 array format (Fig. 2B: after FSL machining, and Fig. 2C: after chemical etching). The multi-chip plate was realized through the aligned bonding of top and bottom PDMS layers, which incorporated a total of 36 reservoirs for media exchange. Each Lumina-Chip had four dedicated wells serving as inlets and outlets for the lateral lumens, in addition to two access ports for filling the central channel with hydrogel (Fig. 1H And Fig. 2D). Three main criteria were considered in the design of the multi-chip plate. First, the spacing between the wells within the same row (9 mm) was determined in accordance with the standard dimensions of multichannel pipettes (Fig. 2D and E). Second, the dimensions of the plate (65 mm × 55 mm) were chosen to match standard 96 mm Petri dishes, which enables sterile culture of the multi-chip inside Petri dishes (Fig. 2D and E). Third, the FSL machining parameters were set to minimize the surface roughness of the channels to allow for optical microscopy (Fig. 2F–H). We conducted surface roughness measurements across five distinct regions on the master mold of the Lumina-Chip, including two on the lateral channels and three within the middle channel (Fig. 2F). The mean roughness depth (Rz), arithmetic mean roughness (Ra), and root mean square roughness (Rq) are small and similar in all regions, except for a notable deviation occurring near the edges of the middle channel, where the surface roughness was observed to be slightly higher (Fig. 2G and H).

**Fig. 2:**
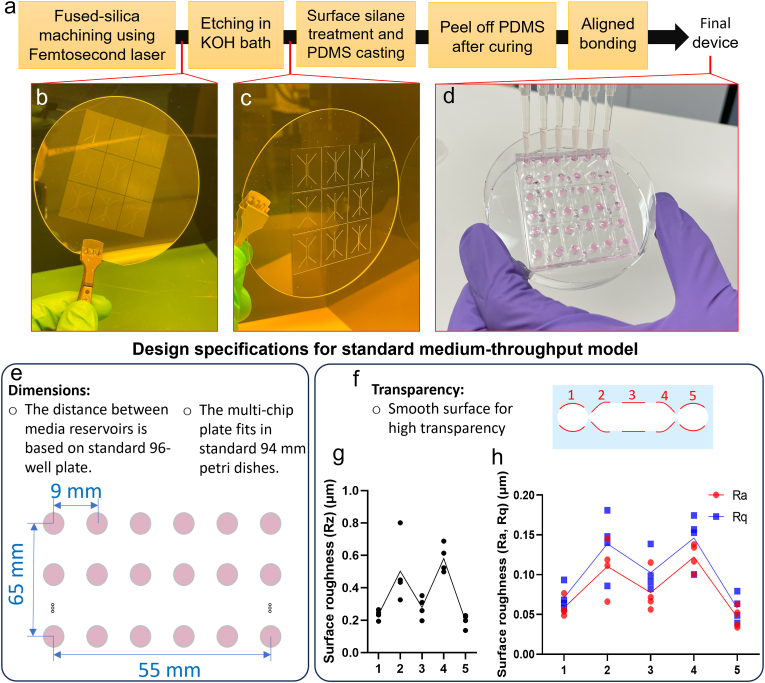
**The fabrication process and design specifications of the medium-throughput Multi-Lumina-Chips model. a**, Fabrication protocol of the Multi-Lumina-Chips model. **b**-**c**, Fused silica glass wafer including the mold features for nine Lumina-Chips, after (**b**) FSL machining, and (**c**) chemical etching. **d**, The final Multi-Lumina-Chips model after fabrication; the reservoirs of the chips are filled with cell medium using a multichannel pipette. **e**, Design specifications of the Multi-Lumina-Chips based on standard 96-well plates and 94 mm Petri dishes. **f**-**h**, Surface roughness measurements at five locations in the Multi-Lumina-Chips; (**f**, **g**) mean roughness depth (Rz), (**f**, **h**) arithmetic mean roughness (Ra), and root mean square roughness (Rq) at five locations (1–5) on the machined surface in the Lumina-Chip. Mean roughness depth (Rz) refers to the average vertical distance between the five highest peaks and the five lowest valleys over a measured length. Arithmetical mean roughness (Ra) is the average of the absolute values of the deviations from the mean surface level across a specified length. Root mean square roughness (Rq) is calculated as the square root of the average of the squared deviations from the mean surface level.

### 2.2 Permeability of the vessel in the Lumina-Chip

To assess the functionality of the endothelialized vessel within the Lumina-Chip, we conducted a permeability experiment utilizing dextran molecules with molecular weights of 10 kDa and 70 kDa (Fig. 3A–E). When the HUVECs fully covered the vessel lumen after 3 days in culture (Fig. 3A), dextran was introduced into the vessel lumen. The 10 kDa dextran permeated the vessel wall and diffused into the collagen matrix (Fig. 3B–E). In contrast, 70 kDa dextran exhibited less permeation through the vessel wall, therefore showing less diffusion into the matrix (Fig. 3C–E), however this difference was not statistically significant (Fig. 1F). The diffusive permeability significantly reduced in the presence of endothelium for both 10 kDa and 70 kDa dextran compared to control experiments without endothelium (Fig. 1F). Also, DMSO treatment of the vessel, known to disrupt the endothelium, resulted in enhanced 70 kDa dextran diffusion and higher diffusive permeability (Fig. 3D–F). To model the permeability dynamics of the vessel over time, we carried out a numerical simulation of the dextran assay, confirming the experimentally observed behavior, as shown in Supplementary Fig. 1.

**Fig. 3:**
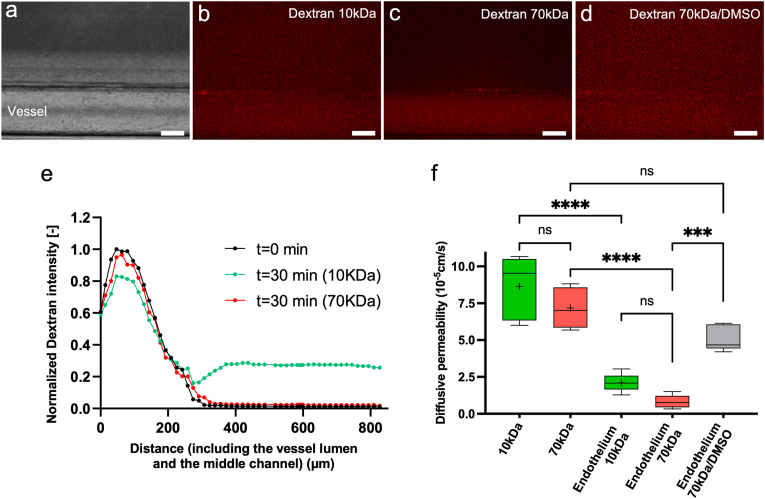
**Permeability analysis of the blood vessel in the Lumina-Chip. a,** Phase contrast image of HUVECs lining the vessel lumen in the Lumina-Chip. **b**-**c**, Fluorescent images of (**b**) 10 kDa and (**c**) 70 kDa dextran 30 min after introducing 25 μg/ml of dextran solution into the vessel lumen. Scale bar, 100 μm **d,** Fluorescent image of 70 kDa dextran in a DMSO treated vessel; the dextran was added to the vessel lumen and reservoirs after 30 min of DMSO treatment, and the fluorescent image was recorded at timepoint 60 min. Scale bar, 100 μm. **e,** The normalized dextran intensity profiles at t = 0 min and t = 30 min for 10 kDa and 70 kDa dextran. **f,** Diffusive permeability of the vessel in the Lumina-Chip for 10 kDa and 70 kDa dextran both in presence and absence of the endothelium, and in a DMSO treated vessel using 70 kDa dextran. ∗∗∗P < 0.001 and ∗∗∗∗P < 0.0001 indicate statistical significance. NS, not significant. One-way ANOVA test was done to determine statistical significance.

### 2.3 Cancer invasion and intravasation in the Lumina-Chip

We utilized the Lumina-Chip to investigate cancer cell invasion and intravasation dynamics (Fig. 4, Fig. 5, Fig. 6, and Supplementary Fig. 2). First, we show invasion behavior of cancer cells in the absence of other cells, but under a FBS gradient (Fig. 4A and Supplementary Fig. 2A and E). MCF-7 or MDA-MB-231 tumoroids, with an average size of 150 μm, were introduced into the right lumen. The middle channel, filled with 2.5 mg/ml collagen I, had a width of 800 μm (Fig. 1J). A higher percentage of fetal bovine serum (FBS)-supplemented media (20 % FBS) was introduced into the left channel, but no endothelial cells were cultured in this channel in initial experiments (Fig. 4A–F). The MCF-7 tumoroids attached to the matrix initially and exhibited growth along the channel (Fig. 4B–D). MCF-7 tumoroids specifically demonstrated luminal growth, occupying the channel surface before showing minimal invasion (Fig. 4E). In contrast to the MCF-7 tumoroids, the MDA-MB-231 tumoroids did not grow within the channel. These invasive tumoroids underwent a transition from their spherical shape, adhering to the matrix along the gap connecting the lumen to the matrix. Subsequently, numerous cells invaded into the ECM (Fig. 4F). Unlike the MCF-7 cells, the majority of MDA-MB-231 cells invaded into the matrix, with only a few remaining in the ductal channel (Fig. 4F). Several of the MDA-MB-231 cells detached from the invasive group and migrated further into the gel (Fig. 4F, white arrows).

**Fig. 4:**
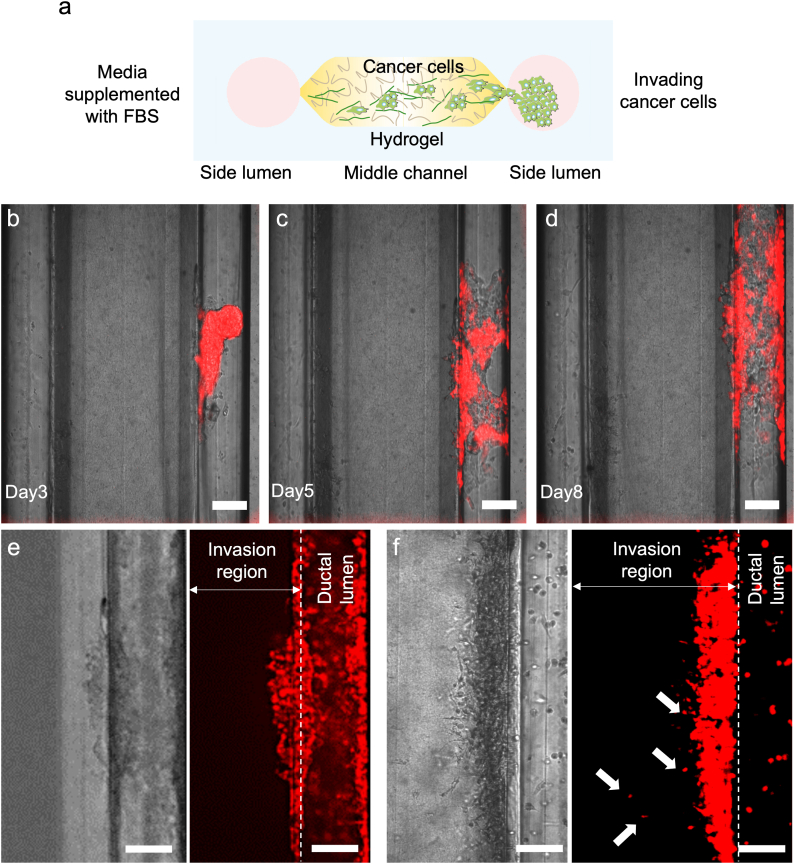
**Cancer invasion in the Lumina-Chip. a**, Schematic representation of the cancer cell invasion in the Lumina-Chip; the media in the left lumen is supplemented with FBS, and the right lumen is filled with cancer tumoroids; the middle channel contains collagen I. **b**-**d**, Labeled MCF-7 tumoroids cultured in the right lumen for 8 days. Scale bar, 200 μm. **e**, Minor collective invasion of labeled MCF-7 cells into the collagen I in the middle channel after lining the ductal lumen (after 11 days in culture). Scale bar, 200 μm. **f**, Several individually labeled MDA-MB-231 cells invading (white arrows) into the collagen matrix after escaping from the invasive group and migrating further into the collagen (after 6 days in culture). Scale bar, 200 μm.

**Fig. 5:**
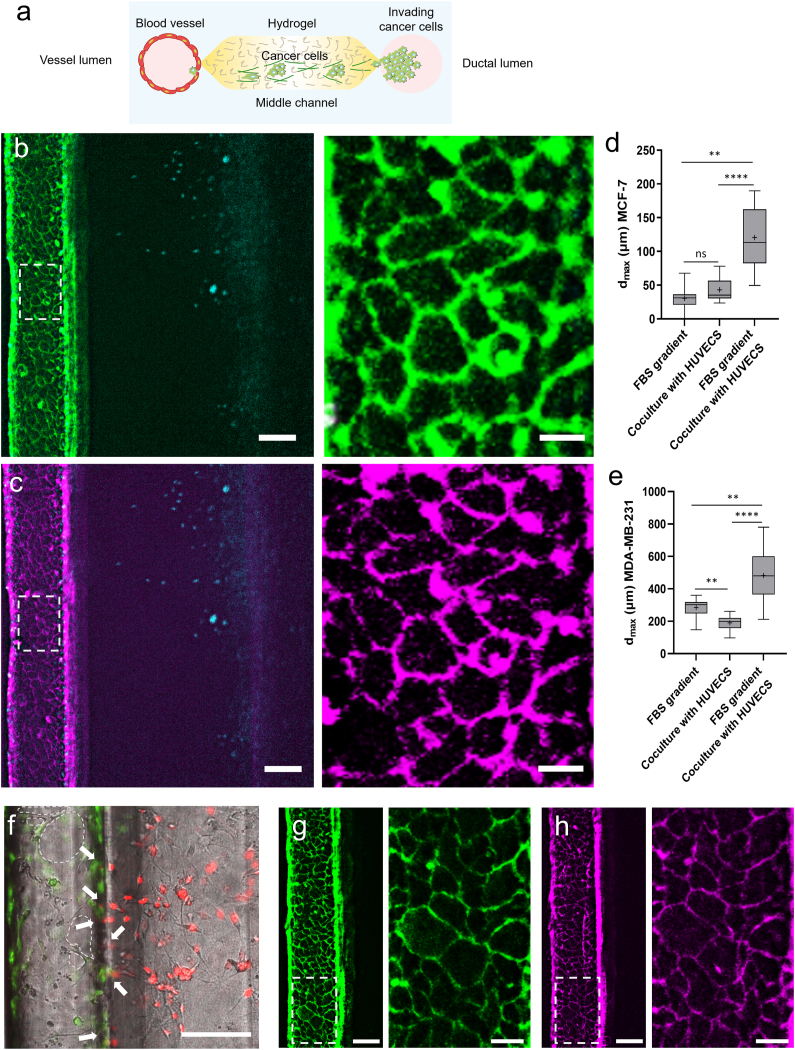
**Cancer invasion and intravasation in the Lumina-Chip. a**, Schematic representation of the cancer cell invasion and intravasation in the Lumina-Chip; the left lumen is lined with endothelial cells, and the right lumen is filled with cancer tumoroids; the middle channel contains collagen I. **b**-**c**, MDA-MB-231 cell invasion from the right lumen into the matrix toward the vessel lumen that is lined with HUVECs. **b** and **c** are alike except that HUVECs are stained for CD31 in **b**, and for VE-cadherin in **c**. CD31 (green), VE-cadherin (magenta) and cell nuclei (cancer cells-DAPI, cyan). Scale bar, 200 μm. Enlarged images show the areas of the dashed rectangles. Scale bar, 40 μm. **d**-**e**, The maximum invasion distance (**dmax**) of (**d**) MCF-7 and (**e**) MDA-MB-231 cells in three conditions: under a FBS gradient without co-culture with a vessel, and with co-culture with the vessel with and without an FBS gradient. These are data obtained from at least three regions of interest per chip, and three chips per condition. **f**, Intravasation of MDA-MB-231 cells (red) into the vessel lined with HUVECs (green). The dashed lines show empty regions of the vessel, not covered by HUVECs. Scale bar, 300 μm. **g**-**h**, The endothelium in the vessel lumen in the absence of the cancer cells. These cells were cultured for the same time period as under the cancer invasion condition (10 days). **g** and **h** are alike except that HUVECs are stained for CD31 in **g**, and for VE-cadherin in **h**. CD31 (green), and VE-cadherin (magenta). Scale bar, 200 μm. Enlarged images show the area of the dashed rectangle. Scale bar, 40 μm. **P** < 0.01 and ∗∗∗∗P < 0.0001 indicate statistical significance. NS, not significant. *t*-test and two-way ANOVA test were done to determine statistical significance.

**Fig. 6:**
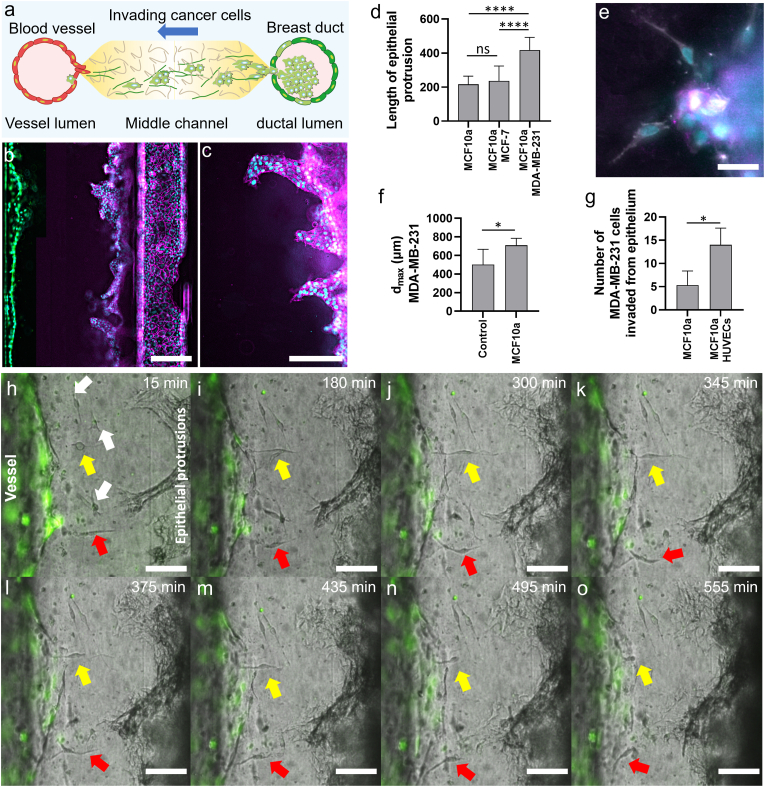
**Invasive ductal carcinoma in the Lumina-Chip. a**, Schematic representation of cancer cell invasion from a breast duct epithelium into the collagen I matrix, and further intravasation into the vessel. **b**, MCF10a cells in the ductal lumen protrude into the collagen I matrix in co-culture with HUVECs, that line the vessel lumen (co-culture of MCF10a and HUVECs). Cell nuclei (cyan), HUVECs (green), and E-cadherin (magenta). Scale bar, 300 μm. **c**, Protrusion of MCF10a cells into the collagen I matrix when co-cultured with MDA-MB-231 cells (co-culture of MCF10a and MDA-MB-231). Cell nuclei (cyan), and E-cadherin (magenta). Scale bar, 100 μm **d**, Length of epithelial protrusions into the collagen matrix in three conditions: MCF10a monoculture, MCF10a/MCF-7 co-culture, and MCF10a/MDA-MB-231 co-culture (Supplementary Fig. 2C**, 2days of MCF10a coverage, and 8 days of co-culture). e,** Invasion of single MDA-MB-231 cells into collagen I from the epithelium layer presenting a filopodia-like morphology (co-culture of MCF10a and MDA-MB-231). Cell nuclei (cyan), CD31 (gray), and E-cadherin (magenta). The MDA-MB-231 cells are stained in gray (CD31-positive), elongating from the epithelial base lined with MCF10a cells stained for E-cadherin (shown in magenta). Scale bar, 20 μm. **f,** The maximum invasion distance dmax of MDA-MB-231 cells in co-culture with MCF10a cells compared to the control (no MCF10a) **(**Supplementary Fig. 2C, 2days of MCF10a coverage, and 8 days of co-culture). **g**, Number of individual MDA-MB-231 cells invaded from the epithelium in triculture with MCF10a in the ductal lumen and HUVECs in the vessel lumen, in comparison with a control (no HUVECs) (Supplementary Fig. 2D and 8 days of tri-culture). **h**-**o**, Images of cancer cell invasion and intravasation progression at several time frames up to 555 min after 6 days in culture (triculture of MCF10a, MDA-MB-231, and HUVECs). Arrows show the cancer cells in the collagen I between the epithelium (right) and endothelium (left); yellow and red arrows in (**h-o**) each point to an individual cancer cell migrating towards and intravasating into the vessel. HUVECs (green). Scale bar, 100 μm. *P* < 0.05 and ∗∗∗∗P < 0.0001 indicate statistical significance. NS, not significant. *t*-test and one-way ANOVA test for (**d**) and *t*-test for (**f**, **g**) were done to determine statistical significance. The data in **d**, **f**-**g** are obtained from at least three regions of interest per chip, and three chips per condition.

To explore the interaction between the blood vessel and tumoroids, HUVECs were seeded in the vessel lumen before introducing tumoroids into the ductal lumen, allowing them to fully cover the lumen's surface, as schematically shown in Fig. 5A and Supplementary Fig. 2B and E. Qualitatively, the cancer tumoroids exhibited similar behavior compared to the condition without endothelial cell culture; MCF-7 cells proliferated within the ductal channel, while MDA-MB-231 cells adhered to the collagen I and invaded into the ECM. Unlike the MCF-7 cells (Supplementary Fig. 3), which displayed minor and collective invasion, MDA-MB-231 single cells invaded the matrix after 3 days in culture (Fig. 5B and C). Throughout this period, the endothelial barrier in the vessel lumen maintained its integrity, as evidenced by the expression of adherens junctions (Fig. 5B and C). The maximum invasion distances of cancer cells were assessed under three conditions: 1) in the presence of an FBS gradient (20 %), but without co-culture with a vessel, 2) co-culture with the vessel, but without an FBS gradient, 3) co-culture with the vessel and an FBS gradient. For both the MCF-7 and MDA-MB-231 cells, the maximum invasion distance was notably highest in the condition of co-culture with the HUVECs in the vessel and the FBS gradient, i.e. culturing HUVECs in the vessel channel significantly increased the maximum invasion distance over the presence of the FBS gradient alone (Fig. 5D and E). Furthermore, MDA-MB-231 cells exhibited a substantially larger invasion distance compared to MCF-7 cells across all conditions. After 8 days of co-culture of tumoroids and the vessel, the MDA-MB-231 cells migrated throughout the complete collagen I chamber towards the vessel, and several cells did intravasate into the vessel through the endothelial barrier (Fig. 5F). When MDA-MB-231 cells were in close vicinity to the vessel, the endothelial cells showed a discontinuous morphology; empty regions were observed that were not covered by cells, suggesting enhanced permeability and leakiness of the vessel (Fig. 5F). On the other hand, when endothelial cells in the vessel lumen were kept in culture for the same period of time, the cellular morphology remained intact, confirmed by the expression of endothelial junctions (Fig. 5G and H).

### 2.4 Cancer invasion from the epithelium and intravasation through the endothelium in the Lumina-Chip

We integrated both a blood vessel and a breast duct within luminal channels of the Lumina-Chip, allowing for the emulation of cancer cell invasion from a breast duct into the ECM channel and subsequent migration towards and intravasation into the vessel lumen (Fig. 6A and Supplementary Fig. 2C–E). When normal epithelial cells (MCF10a) were seeded in one channel and HUVECs in the other, both cell types proliferated and covered the lumens within 3 days. MCF10a cells protruded from the breast duct into the collagen I (2.5 mg/ml), while preserving E-cadherin cell-cell junctions (Fig. 6B). Simultaneously, HUVECs lining the collagen I at the interface with the vessel lumen deformed the collagen boundary and displayed sprouting (Fig. 6B). When MDA-MB-231 breast cancer cells were introduced into the breast ductal lumen in the absence of ECs in the vessel lumen (Supplementary Fig. 2C), and the chip was maintained in culture, a notable elongation of the epithelial protrusions was observed (Fig. 6C). The length of protrusions was similar for the MCF10a/MCF-7 co-culture and the control (MCF10a); however, in the MCF10a/MDA-MB-231 co-culture, the protrusions were twice as long as observed in the MCF10a/MCF-7 co-culture (Fig. 6D). MDA-MB-231 cancer cells invaded through these protrusions, exhibiting a mesenchymal morphology as they exited the epithelium (Fig. 6E). Moreover, the maximum invasion distance (*d*_max_) of the MDA-MB-231 cancer cells into the collagen I increased when they were co-cultured with MCF10a cells compared to the mono-culture of MDA-MB-231 cells in the ductal lumen (Fig. 6F).

To explore the influence of endothelial cells on the invasive behavior of MDA-MB-231 cancer cells from the epithelium, HUVECs were seeded to line the vessel lumen (Supplementary Fig. 2D). Consequently, the number of invading MDA-MB-231 cells from the epithelium into the collagen I ECM increased significantly (Fig. 6G). During a 6-day cell culture, the cancer cells invaded and migrated further into the collagen I between the epithelial protrusions from the breast duct and the endothelium in the vessel; the arrows in Fig. 6H–O indicate the positions of cancer cells. Some cancer cells exhibited elongation before intravasation, displaying a mesenchymal morphology (yellow and red arrows in Fig. 5H–O each point to an individual cancer cell migrating towards and intravasating into the vessel). Upon contact with the endothelium, ECs facilitated a gateway for cancer cells to traverse from the ECM, intravasating into the vessel lumen (Fig. 6J–O and Supplementary Video 1).

## 3 Discussion

We introduced femtosecond laser (FSL) machining as a method to realize OoC devices with tubular channels. This manufacturing approach offers unique possibilities to satisfy the combination of diverse desired criteria for the versatile fabrication of OoC systems. The main advantage of femtosecond lasers in comparison with conventional lasers is the minimized thermal damage during machining due to the ultra-short pulses. This results in very little heat transfer to surrounding material. This advantage is ideal for higher precision and clean cuts in micromachining and microsurgery. Moreover, in contrast to conventional lasers, with a femtosecond laser it is possible to delicately modify transparent materials like fused silica both on the surface as well as in the interior of the material. Due to these properties, FSL machining enables us to make precise molds in glass with submicron resolution and with a very large design freedom, allowing complex shapes (curved, round, bent, cornered), with a wide range of dimensions (microns to millimeters), on large surface areas (tens of cm^2^) and in relatively thick substrates (millimeters). Such design freedom cannot be achieved using photolithography. FSL machining can also be used to directly structure other materials, such as hydrogels [27]. Furthermore, using the FSL machined product as a mold, or transferring it to a replicated mold (such as in epoxy, as shown in this study), enables the repetitive creation of OoC devices using casting, (injection) molding, or hot embossing [41]. This replica molding approach is suitable for a large range of different materials, from PDMS (as we show in this study) to thermoplastic polymers, and the technique may also be used for casting hydrogels. These advantages together make this approach not only robust but also scalable in terms of higher-throughput production with excellent reproducibility.

As a showcase study, we used the FSL technique to create a new version of the most common type of compartmentalized OoC devices (made by the group of R. Kamm at MIT), with tubular channels as side lumens at either side of a middle compartment instead of rectangular channels: the Lumina-Chip. The 300 μm diameter size of the tubular channels was chosen based on common OoC systems that often include engineered channels in the range of several hundreds of microns. The interfaces between the side lumens and the hydrogel contained in the middle compartment are formed by a gap or slit of 30 μm that is fully open along the entire length of the channels. This is an improvement over previous compartmentalized OoC models that often include pillars for separating neighboring channels and for keeping hydrogel contained within separate channels. These separating pillars may affect the morphology and behavior of cells at the interface between the neighboring channels. Other OoC models utilize phase guide steps to keep hydrogel confined in one channel neighboring another one [42]; unlike the separating posts, the phase guide model causes less disturbance to cells at the interface. However, current approaches using phase guides do not allow for controlled curvature of the interface, needed for mimicking a tubular geometry. Increasing the size of the gap between the tubular lumens and the middle channel in the Lumina-Chip is possible; the choice of 30 μm allows for maintaining the tubular geometry while leaving enough space for cell passage and migration. Furthermore, the induced surface tension on the hydrogel surface prior to cross-linking is sufficient to keep the hydrogel contained within the middle compartment.

Using FSL mold machining combined with replica molding, we fabricated a multi-Lumina-Chip device composed of nine chips. The distance between media reservoirs in the device is compatible with the 96-well plate standard and with multichannel pipetting; the dimensions of our multi-chip plate were chosen to fit standard 94 mm Petri dishes (Fig. 2E). This design choice was made to ensure the maintenance of optimal humidity levels during the culture period within the incubator by inserting the plate in a closed Petri dish, thereby mitigating the risk of rapid media evaporation from the reservoirs. However, it is possible to enlarge our multi-chip plate to a standard 96-well-plate format (127.8 mm × 85.5 mm), on which 16 independent Lumina-Chip models can fit, enabling higher-throughput testing. Still, high-throughput multi-chip models will not be fully compatible with 96-well plates because of the screening region where the channels locate between reservoirs. Hence, 25 % of the space will be occupied by channels rather than reservoirs. There is ongoing work on defining and realizing standardized OoC platforms, which include micro-pumps to generate flow [43]. These will take up some space as well. However, precise design specifications or miniaturizing the fluidic components can facilitate the fabrication of standardized high-throughput models. FSL machining introduces roughness on the glass mold and consequently on the surfaces of the PDMS chip. A smooth surface with minimal roughness is preferred to reduce light scattering and distortion for cell imaging. We performed a contact profilometry experiment on the engraved surfaces in different regions of the chip, resulting in quantification of the surface roughness (Fig. 2F–H). We did this test to ensure that the surface is smooth enough to not interfere with (fluorescent) microscopy and cell attachment. An average PDMS surface roughness (Ra) ranging from a few nanometers (nm) to tens of nanometers is desirable for cell adhesion and fluorescent microscopy [44]. We measured a mean roughness Ra below 100 nm in the luminal regions, and this range was permissive for cell adhesion (Fig. 2H). These values also support the submicron resolution of femtosecond laser machining. The roughness in all regions enabled optical microscopy. Moreover, the surface roughness did not affect the PDMS peel-off in the demolding process.

We characterized the barrier function of the vessel walls within the Lumina-Chip by permeability experiments using dextran molecules of different molecular weights (10 and 70 kDa), showing differential permeation through the vessel wall (Fig. 3A–E) [32]; smaller molecules exhibited enhanced permeation, whereas larger molecules showed less permeation, however this trend was not found to be statistically significant (Fig. 3F). The barrier integrity was confirmed via fluorescent images of CD31 and VE-cadherin, which expose intact adherens junctions (Fig. 5G and H). Moreover, the diffusive permeability into the matrix in the absence of endothelium, as a control, showed significantly higher permeability values for both 10 and 70 kDa dextran compared to the endothelium-present condition (Fig. 3F). Furthermore, when the vessel integrity was disrupted by pre-treatment with DMSO, permeability significantly increased for 70 kDa dextran, providing additional proof of the endothelium's integrity (Fig. 3D–F). These experiments, including controls (no endothelium and DMSO) (Fig. 3), and endothelial junction staining (Fig. 5G and H), substantiate the presence of the endothelium. We note that absolute permeability values can vary significantly between studies due to differences in experimental parameters such as dextran type, cell line, matrix composition, and system geometry [22,32,45]. Although our study does not aim to standardize permeability assays across organ-on-chip systems, as recently addressed in detail by Nahon et al. [46], we highlight this variability to raise awareness of how subtle differences in experimental setups can influence absolute measurement values. Despite these discrepancies, relative comparisons (e.g., between dextran sizes or treatment conditions within the same system) remain robust and informative.

The simulations of the diffusive permeability within the Lumina-Chip agree qualitatively with the experiments, and the results reveal the spatial distribution and time evolution of the diffusion within the middle channel, which is hard to experimentally visualize in the cross-section of the Lumina-Chip (Supplementary Fig. 1). The simulations provide more insight into the spatial distribution over time of the diffusion of a molecule through and beyond the endothelium. More importantly, simulations enable us to model different experimental scenarios that may be harder to establish in experiments. For example, diffusion through hydrogels with different porosity, and/or different flow conditions, can be modeled to predict or guide experiments. Supplementary Fig. 4 showcases different possibilities. Supplementary Fig. 4A shows the diffusive permeability in matrices with three different diffusive coefficients. Connecting to experiments, this value can differ for different types of hydrogels and for varying concentrations [[47], [48], [49], [50]]. Supplementary Fig. 4B shows the scenario when an additional source of the diffusing molecule is added in the right channel, in this case at half of the initial intensity as in the left channel. This condition resulted in substantially different diffusion profiles within the matrix. In addition to these concrete examples, many other scenarios can be simulated using our numerical model.

We designed the Lumina-Chip for including vessels and epithelial ducts within the same system. As a showcase study, we focused on emulating the breast epithelial duct, next to a blood vessel, to mimic breast cancer metastasis in the chip (Fig. 4, Fig. 5, Fig. 6 and Supplementary Fig. 2). Two different breast cancer cell types, namely MCF-7, as non-invasive, and MDA-MB-231, as invasive cell lines, were utilized in our model to investigate the dynamics of cancer invasion. While MCF-7 cells primarily demonstrated luminal growth within the ductal lumen, representative of Ductal Carcinoma In Situ (DCIS), MDA-MB-231 cells displayed invasive behavior, transitioning from a spheroidal shape to individual cells invading into the ECM (Fig. 4E and F). The majority of MDA-MB-231 cells invaded and just some cells remained in the ductal channel (Fig. 4F); several of these cells detached from the invasive group and invaded further into the collagen I (Fig. 4F - white arrows).

Since it is already well-established that a serum-enriched environment can induce cancer invasion and that a serum gradient influences the invasion direction [[51], [52], [53]], we included FBS in the opposite lumen for some of the cases in our invasion study. Interestingly, adding HUVECs to the opposite lumen in the presence of FBS substantially increased the maximum invasion distance. HUVECs in the absence of FBS did not induce a strong invasive response (Fig. 5D and E). It is known that the presence of FBS can influence the HUVEC morphology and signaling pathways [54,55]. For example, the findings of Russell et al. showed that serum-deprivation induces superoxide production and proinflammatory responses in HUVECs [55]. We speculate that our finding regarding the interaction between enriched-FBS medium and HUVECs bringing about increased cancer invasion distance could be caused by a series of events; serum-enriched medium lowers the proliferation rate and induces expressions that might send signals influencing the cancer cell migration [54]. However, testing this hypothesis needs further investigation into the molecular interaction between the vessel and cancer cells in different medium conditions.

In this study, for the first time on a single chip, we successfully emulated the combined initial phases of metastasis, i.e. cancer invasion from the epithelium, followed by migration towards the endothelium, and intravasation into the vessel. In vitro models modeling the initial step within the cancer metastasis cascade, invasion, are often overly simplified. Cancer invasion in vivo is a complex process, but nevertheless, having a basic model can enable studies for getting better understanding of mechanisms in metastasis. The general consensus is that in the first steps of cancer growth, the epithelial cells alter their phenotype, undergo epithelial to mesenchymal transition (EMT) [56], turn into cancer cells, and proliferate nonstop. The cancer cells might directly invade into the surrounding matrix, but the invasion can also occur away from the main tumor mass, i.e. at another transversal location in the breast duct [57,58]. In the latter case, these cancer cells proliferate and fill the ductal lumen, leading to the condition known as Ductal Carcinoma In Situ (DCIS) [59]. The tumor can also expand and migrate intraductally [60,61]. Subsequently, the cancer cells may invade from various locations within the duct, which is still lined by epithelial cells supported by an intact basal lamina [62]. The basement membrane, typically associated with the epithelial layer, plays a crucial role in this context [37,58,62], and the mechanisms by which cancer cells breach this structure remain relatively underexplored.

Many studies focused on cancer invasion within a matrix, but to study invasion in composite environments closer to in vivo environments, more advanced models are necessary. In a recent study, we generated a heterogeneous ECM model by encapsulating cancer cells in Matrigel droplets, embedded in collagen I to mimic cancer cell invasion through a basement membrane; however, the presence of normal epithelial cells as the initial cancer invasion barrier was still lacking [38]. Another component that is important for studying metastasis is the vasculature. Several CoC models include the vasculature based on self-assembly of ECs into vessel networks [36,63]. The nature of this process leads to random vascular network shapes, limiting their reproducibility for cancer studies such as invasion/migration assays. Moreover, in this approach, cancer cells need to be mixed with ECs in the gel prior to network formation, which may not be desirable for specific cancer research, especially when sequential introduction of cells and chemical compounds is necessary.

In the present study, we aimed to emulate invasive ductal carcinoma (IDC) by mimicking a breast duct lumen lined by normal breast epithelial (MCF10a) cells, and then introducing breast cancer tumoroids within the duct (Supplementary Fig. 2C and D). MCF10a cells showed protrusive behavior in collagen I (2.5 mg/ml) (Fig. 6A–C); this is in accordance with previous observations of branching/protrusion behavior of MCF10a cells [38,64]. The length of MCF10a protrusions was not affected by the presence of MCF-7 cells, however, it increased two-fold in co-culture with MDA-MB-231 cancer cells (Fig. 6D). The MDA-MB-231 cells did pass the epithelium and invaded into the collagen I matrix; when we increased the collagen I concentration to 5 mg/ml, the single MDA-MB-231 cells were often located at the tip of the protrusions into the matrix (Supplementary Fig. 5). This highly motile behavior and mesenchymal phenotype of MDA-MB-231 cells may well be the reason for them pushing the MCF10a protrusions forward into the matrix, and then invading into the matrix as leader cells (Fig. 6E). Interestingly, we observed an increase in the maximum invasion distance d_max_ of MDA-MB-231 cells when they were cultured in breast ducts formed by MCF10a cells (Fig. 6F) compared to the control situation without MCF10a cells. Hypothetically, the MCF10a protrusions, generated prior to cancer tumoroid introduction, may be used as tracks by MDA-MB-231 cells to facilitate their migration in comparison with migrating through pure collagen I (2.5 mg/ml). Here, we show that interaction between invasive cancer cells and normal epithelial cells in the ductal channel can influence cancer invasion, which also depends on ECM concentration. Moreover, including HUVECs in the system to form a blood vessel in the lumen opposite to the ductal lumen substantially increased the number of MDA-MB-231 cells invading from the epithelium (Fig. 6G), underlining the importance of the inclusion of all main cellular components and their interactions in vitro models for studying cancer cell invasion and intravasation. In the experiments performed with the tumoroid-breast duct-vessel triculture, we did capture the first stages of MDA-MB-231 cell intravasation into the vessel. Upon cancer cell migration towards and subsequent contact with the endothelium, we observed opening of the space between two endothelial cells to facilitate cancer cell intravasation (Fig. 6H–O and Supplementary Video 1).

Our current study primarily focuses on demonstrating the technical feasibility of our Lumina-chip, and showcasing its possibilities to create and study epithelial-endothelial tubular structures through cancer invasion and intravasation experiments, which show that the interactions between different cells in different (tubular) compartments which our device enables are highly relevant. However, although not in scope in the present study, we acknowledge the importance of analyzing functional cellular responses and interactions in more depth to further enhance our understanding of the biological mechanisms and implications of our observations. In particular, future work will include further phenotypical characterization for example through live imaging of specific markers like vimentin or EpCAM, indicating (changes in) mesenchymal and epithelial phenotype of cancer cells. In addition, time-dependent analysis of soluble factors and gene and/or protein changes will be performed. Although such analyses were not carried out in the present study, the design and compartmental nature of the Lumina-chip allows for the regular collection of media and cells from the different channels separately at different timepoints, enabling such assays. Similarly, further investigation into the phenotypical status of the epithelial and endothelial cell populations, specifically in terms of barrier integrity and growth factor production over time, will be essential. We observed morphological changes and varying integrity, suggesting dynamic cell–cell communication, potentially through both soluble and contact-mediated mechanisms. These aspects are noted as important directions for future studies, particularly if the device will be extended to patient-derived models.

We have shown that FSL machining can be utilized to include tubular channels in OoC systems and, as an example, we created the Lumina-Chip to study invasive ductal carcinoma by including ductal epithelial tissues and blood vessels within a single chip. Even though this model provides advantages, as well as being user-friendly and suitable for upgrading to higher throughput, it has its own limitations. First, the channels are not completely surrounded by hydrogel like in vivo, but only along the gaps between the side lumens and the middle channel; therefore, the full interaction between the lumens and the ECM cannot be studied (for example epithelial inflammation or vasodilation). It is however possible to include hydrogel coatings or layers in the channels, for example using approaches from some recent studies in which conventional techniques (such as sacrificial molding or viscous finger patterning) were used to generate hydrogel-based channels inside PDMS-based channels [63,65]. In the future, we will use the FSL technique to directly structure hydrogels and generate multiple and/or interconnected lumens within different types of (synthetic) hydrogels. The channels that are obtained in hydrogels using other methods have been very beneficial for replicating biological phenomena; using FSL machining will enhance the precision of the features over a broad range of dimensions. However, the higher-throughput production of devices, as shown with the replica molding presented in the current paper, will be lost. Second, it is known that shear stress induced by flow affects endothelial vessel properties [66]. The only shear stress applied in our experiments is through media refreshment, which may also influence the vessel integrity, but it does not cause physiological shear stresses. This temporarily generated maximum shear stress of ∼0.736 Pa does not properly mimic the continuous flow in vivo (see Materials and Methods). It is known that applying different flow conditions (e.g., constant unidirectional, bidirectional, or pulsatile) will affect the morphology, alignment, and function, especially of the endothelial cells lining the blood vessel lumen [3,[67], [68], [69], [70]]. The design of the Lumina-Chip does allow for inducing and controlling flow through the lumens if they are connected to appropriate pumps; this possibility will be included in future work.

## 4 Conclusion

We used an innovative fabrication approach based on FSL machining to realize curved features in microfluidic chips. We utilized this technique to fabricate the Lumina-Chip, a chip containing two channels with circular cross-sections, or tubular lumens, both connected along their entire length to a larger middle channel. This unique architecture enabled us to integrate a blood vessel by including endothelial cells in one lumen, as well as a breast duct, by including epithelial cells in the other lumen, separated by ECM formed by collagen I in the middle channel, reproducing the in vivo organization of these structures. Permeability analysis showed that the vessel wall in the Lumina-Chip had good barrier functionality. These results indicate that our round channels provide a better physiological representation of tubular structures in vivo. After introducing tumoroids in the breast duct in our chip, we could observe cancer cell invasion into the ECM from epithelial protrusions, migration towards the blood vessel, and intravasation of cancer cells into the vessel, with different behaviors for different cancer cells and for different experimental conditions. As far as we know, this is the first time that these first stages of cancer metastasis are observed in a single chip. Since the fabrication process is based on making a mold from which many chips can be replicated, it can be used for medium-throughput manufacturing and subsequent experimentation. These results suggest that our Lumina-Chip is a valuable platform that forms a good basis to create and study organ and disease models containing tubular structures in general. The microfluidics architecture of the chips enables to apply controlled flow, which will be the focus of our upcoming research.

## 5 Methods

### 5.1 Cell culture

HUVEC, MCF10a, MCF-7, and MDA-MB-231 cells were utilized in our experiments. The cell passage procedure was standardized across these cell types, with minor deviations noted where applicable. Cells were expanded in T75 cell culture flasks at 37 °C and 5 % CO_2_, and cultured in relevant culture medium. HUVECs were cultured in endothelial cell growth basal medium 2 (Promocell, C-22211) including supplements (Promocell, C-39211); the culture medium of MCF10a cells contained DMEM-F12 (Merck, D0697) supplemented with 20 ng/ml human epidermal growth factor (hEGF) (Merck, 6225363-8), 0.5 μl/ml Hydrocortisone (Merck, 50-23-7), 100 ng/ml cholera toxin (Merck, 9012639), 10 μg/ml insulin (Merck, 11061680), 5 % horse serum (Merck, H1270) and 1 vol% Penicillin/Streptomycin Solution (P/S) (Sanbio, SCC0503)); MCF-7 and MDA-MB-231 cells were cultured in RPMI-1640 medium (Gibco, 61870010), supplemented with 10 vol% Fetal Bovine Serum (FBS) (Bovogen, SFBS) and 1 vol% Penicillin/Streptomycin Solution (P/S) (Sanbio, SCC0503). Medium was refreshed every 2–3 days. Cells were harvested with 0.05 % Trypsin/EDTA (except for HUVECS that were harvested using accutase (Merck, SCR005)) and centrifuged at 900 rpm (revolutions per minute) for 5 min. Cells were suspended at the desired density to be used in experiments; HUVECs and MCF10a cells were used in 4⋅10^6^ cells/mL, MCF-7 and MDA-MB-231 in 5⋅10^5^ cells/mL. HUVECS were used between passages 4 and 6, and MCF10a cells between passages 5 and 15.

### 5.2 Generation of reporter cell lines

To generate stable fluorescent cell lines and GFP-HUVECs, lentivirus was produced and cells were transduced and selected for resistance to puromycin as described before [71]. Lentivirus was produced in HEK293T cells by PEI mediated transfection of plasmids pMD2.G, pCMVR8.74 and one of the transfer plasmids: pLenti-PGK:APEX2-NES-mKO2, pLenti-PGK:H2B-mKO2, or pLKO.1 CMV:GFP. Production supernatant was either applied to target cells directly or harvested and concentrated by centrifugation at 50.000*g* at 4 °C for 2 h and aliquoted for storage at −80 °C.

### 5.3 Generation of tumor spheroids

MCF7 and MDA-MB-231 cells were suspended at 10000 cells/ml in the cell media (RPMI-1640 (Gibco, 61870010), supplemented with 10 vol% Fetal Bovine Serum (FBS) (Bovogen, SFBS) and 1 vol% Penicillin/Streptomycin Solution (P/S) (Sanbio, SCC0503)) supplemented with MethoCel (Merck, 94378). Concentrations of 20 % and 2 % stock MethoCel (1.2 % w/v) were utilized for the MDA-MB-231 and MCF-7 cells, respectively. For MDA-MB-231 cells, the media was supplemented with 5 % Matrigel (Corning, CLS356230). 200 μl of the suspension solution was deposited in each well of a round-bottom 96 well-plate (Merck, M3562), and incubated for 24h to generate tumoroids. 10000 cells/ml concentration led to the tumoroids with dimensions of 100–200 μm. The tumoroids were all transferred to a 2 ml eppendorf tube by aspirating 20 μl from the bottom of each well, to be used in invasion experiments.

### 5.4 Master mold fabrication using femtosecond laser machining, and replica molding

The master mold of the luminal channels was made using FSL machining. The process is illustrated in Fig. 1A and consisted of the following steps: i. The patterns were designed in Alphacam software and patterned on a fused silica substrate (SIEGERT WAFER GmbH) using a femtosecond-laser system (f200 aHead, FEMTOprint SA, Switzerland). The laser machining of the Lumina-Chip mold took 8 h, and for the Multi-Lumina-Chip mold, which was composed of 9 chips, the machining took around 72 h. This concerns the period for the entire machining including the channel features and the surrounding area. The machining time for the channel features (including the lateral channels and the middle chambers without the surrounding area) in the Lumina-chip was approximately 100 min. We used a 1 mm thick silica glass, but we only machined in the top 150 μ m layer. ii. The modified patterns within the fused silica wafer were etched in 45 % potassium hydroxide solution for 9 h (91 h for the multi-chip plate). iii. The wafer was rinsed with isopropyl alcohol and ethanol (99 %) and dried with an air jet. iv. The features on the wafer were treated with trichloro(1H,1H,2H,2H-perfluorooctyl) silane to reduce adhesion for molding PDMS. For the production of a large number of chips, an epoxy molding process was used to replicate the master mold. The epoxy replica molding process is explained in detail previously [40]. Briefly: i. PDMS (Sylgard® 184 base with curing agent (both from Merck) at a 10:1 w/w ratio) was poured on the negative fused silica mold and cured at 65 °C for at least 2 h. ii. The PDMS slab was peeled off gently. iii. The epoxy mixture was poured on the positive PDMS features and cured for 3 h at 120 °C. iv. The PDMS slab was peeled off from the epoxy, and the epoxy was used repeatedly for device fabrication.

### 5.5 Lumina-Chip, and multi-chip plate fabrication

The chip was made from two PDMS layers that were bonded to each other. The fabrication process was as follows: i. PDMS (Sylgard® 184 base with curing agent (both from Merck) at a 10:1 w/w ratio) was cast on the epoxy molds, degassed for 15 min, and cured at 65 °C for at least 2 h; the poured PDMS weight was measured and dosed upfront to obtain a final layer thickness of 1.2 mm for the bottom layer and 5 mm for the top layer. ii. The polymerized PDMS slabs were peeled off, and the PDMS edges were trimmed using a cutter. iii. To punch in- and outlet access holes through the top layer, 5 mm biopsy punchers (KAI biopsy punch 4560146922619) were used for the lateral lumens, and 3 mm punchers (VWR, WHATWB100074) were used for the middle channel. iv. The channels were sealed by aligned bonding of the top and bottom PDMS layers; the PDMS slabs (features facing up) first were both exposed to 20 W air plasma for 30 s using a plasma asher (Emitech, K1050X). v. The bottom layer was immediately placed under a microscope, and a droplet of ethanol (25 μL) was dispensed on the features; the top layer was placed on the bottom layer; the ethanol in between enabled sliding layers for alignment. vi. The chip was heated at 65 °C for at least 1 h for ethanol evaporation and final bonding. Multi-chip plate fabrication was similar to individual Lumina-Chip fabrication, with a change in the aligned bonding step. Specifically, a droplet of ethanol per chip within the multi-chip plate was carefully dispensed, and features of the chips, especially those located at the corners, were aligned using a stereoscope. The plate was left undisturbed to allow for ethanol evaporation at room temperature. To achieve full bonding, the assembled complex underwent a thermal treatment at 65 °C for a minimum duration of 1 h. This procedure ensured the thorough cohesion and integrity of the multi-chip plate, rendering it suitable for subsequent experiments.

### 5.6 Height and surface roughness measurements

The Bruker Dektak XT contact profilometer was utilized to measure the height and the surface roughness of patterned features of the fused silica glass master-mold. This profilometer operates based on the deflection of a fine stylus that raster-scans over features ranging in height from 1 mm down to 5 nm. A 50 nm probe was used for surface roughness measurements, with measurements repeated along several lines on the surface of the fused silica mold, perpendicular to the main axes of the channels. ProfilmOnline software was used to calculate the line roughness data (ISO4287 Amplitude), including: mean roughness depth (Rz), arithmetical mean roughness (Ra) and root mean square roughness (Rq). Mean roughness depth (Rz) refers to the average vertical distance between the five highest peaks and the five lowest valleys over a measured length, giving a measure of the most prominent surface features. Arithmetical mean roughness (Ra) is the average of the absolute values of the deviations from the mean surface level across a specified length, offering a general measure of surface texture. Root mean square roughness (Rq) is calculated as the square root of the average of the squared deviations from the mean surface level, and it gives slightly more emphasis to larger deviations compared to Ra.

### 5.7 Lumina-Chip preparation and loading

The chips were autoclaved for sterility and all the next steps were conducted in a safety cabinet. The chips were located in a petri dish (94 mm) for incubation, and a smaller petri dish (35 mm), filled with phosphate buffered solution (PBS) was placed next to the chip to increase air humidity. The middle channel of the chip was filled with 0.01 % w/v dopamine hydrochloride (Fisher Scientific, 11409043) overnight to enhance the hydrogel to PDMS attachment. The dopamine hydrochloride solution was aspirated out and the middle channel was washed via introduction and aspiration of PBS. Hydrogel loading: Next, 40 μl of collagen I (2.5 mg ml^−1^) was loaded in the middle channel by pipetting from the inlet, and it was allowed to polymerize in a 37 °C incubator for 30 min. The side lumens were coated with 20 μl of either Matrigel (5 % v/v) for epithelial cells, or fibronectin (20 μg/ml, Merck, F2006-1 MG) for endothelial cells. The coating solutions were aspirated out.

Coating and cell seeding: Next, 20 μl (4e6/ml) of HUVECs or MCF10a cells were seeded in the side lumens and incubated at 37 °C for at least 30 min to facilitate attachment to the luminal base. To ensure proper cell adhesion to the side surfaces of the lumens, the chips were sequentially rotated 90° to each side for 30-min intervals. After incubation, culture medium was placed into the side lumens and medium reservoirs to flush out the non-attached cells. Medium was changed every day for the endothelial lumen and every other day for the breast duct lumen. Media was removed from all four side reservoirs and 100 μl of fresh media was added to the reservoirs on one side of the lumens. Media flowed through the lumens to the other reservoirs due to hydrostatic pressure. Different culture conditions, such as tumoroid, tumoroid-vessel, breast duct, breast duct-vessel, and tumoroid in breast duct-vessel were conducted. For time intervals and further details of the tests performed, see Supplementary Fig. 2.

### 5.8 Wall shear stress calculation for the media exchange

The maximum induced wall shear stress on the tubular channel wall due to the media refreshment was calculated using [72]:

$$\tau_{\max,tubular}=-\frac{\Delta P\cdot R}{2L}$$

where *ΔP* is the pressure drop between the inlet and outlet of the channel at the moment of adding media to the inlet reservoir, *R* is the radius of the tubular channel, and *L* is the length of the channel. We assumed that the flow is fully developed and laminar. The pressure-drop generated by media refreshment was calculated by:

$$\Delta P=\rho gh_{M}$$

where *ρ* is the density of the cell media (assumed as 1000 kg/m^3^), *g* is the acceleration due to gravity (approximately 9.81 m/s^2^), and *h*_*M*_ is the height difference between the liquid levels at the reservoir and the outlet of the tube.

### 5.9 Vessel permeability assay

When the HUVECs fully covered the vessel lumen in Lumina-Chip (after 3 days of culture), 25 μg/ml dextran (Fluorescein isothiocyanate–FITC dextran 10 kDa (Merck FD10s) or 70 kDa (Merck FD70s)) was added to the inlet reservoir of the vessel lumen, filling the lumen to the outlet reservoir. Possible interstitial flow was avoided as much as possible by introducing a low volume of dextran solution into the inlet of the lumen, while adding an equal volume of media (without dextran) to the right lumen. For permeabilizing the vessel wall, 2 % DMSO was infused into the vessel for 20 min prior to the dextran (70 kDa) assay. The fluorescent images of dextran in the vessel lumen and the collagen I channel were taken in time. The fluorescent intensity of dextran was measured in Fiji. Solute transport was measured over 30 min. Permeability coefficients were calculated using the following equation [22,73]:

$$P=\left(\frac{1}{{\Delta I}_{0}}\right)\left(\frac{{TI}_{f}-{TI}_{0}}{t_{f}-t_{0}}\right)\left(\frac{fD}{4}\right)$$

where *ΔI*_*0*_ is the initial step increase in fluorescence intensity across the lumen wall, *TI*_*0*_ is the total initial fluorescence intensity along the line starting from the edge of the vessel and going outwards at the beginning of the assay, *TI*_*f*_ is the total fluorescence intensity along the line starting from the edge of the vessel and going outwards at 15 min, *t*_*0*_ is the initial time point, *t*_*f*_ is the final time point of 15 min, *D* is vessel diameter and *f* is the fraction of the vessel perimeter that is exposed to the diffusive matrix. For calculating ΔI_0_, it is assumed that the concentration difference of dextran across the lumen wall at the beginning of the experiments equals the solute concentration in the lumen.

### 5.10 Simulation of chemical gradient

A finite element (FE) model was developed utilizing COMSOL Multiphysics software to understand and analyze mass transport phenomena within the Lumina-Chip. The model configuration is based on the experimental setup, incorporating two side channels and the middle hydrogel channel. The transport equation of diluted species in the system reads:

$$\partial C_{i}/\partial t+\vec{u}\;.\;{\nabla C}_{i}=\nabla .\left({D_{i}\nabla C}_{i}\right)+R_{i}$$

where *C*_*i*_ and *D*_*i*_ are the concentration and the diffusion coefficient of species *i* respectively, and *t*, $\vec{u}$, and *R*_*i*_ represent time, the fluid velocity vector, and the reaction term of species *i* (which is equal to zero here) respectively. The diffusion coefficient values are provided via vessel permeability assay. The diffusion coefficients for the simulations including different concentrations of hydrogels were assumed half (high) and twice (low) the moderate concentration conditions. For a specific type of hydrogel and concentration, this value can be extracted from the literature [[47], [48], [49], [50]]. The channel and reservoirs are filled with dextran; the dextran solution volume in the reservoirs is larger than in the channel, which compensates for the diffused dextran in the channel. To account for the influence of the dextran in the reservoir, a very slow creeping flow was introduced (Reynolds number <1) in the direction of the main axis of the luminal channel with an initial normalized concentration of 1. Additionally, in order to simulate the permeation between the different zones, a partition boundary condition was employed. This allowed for the establishment of distinct concentrations while maintaining a constant flux:

$$k_{i}=\frac{C_{\text{upstream}}}{C_{\text{downstream}}}$$

in which the coefficients for the 10 kDa and 70 kDa dextrans were set to 0.85 and 0.05 respectively by fitting to the experimental permeability result. The computational mesh (grid) was created using the COMSOL Meshing module. After mesh-sensitivity analysis, 250k tetrahedral elements with an average element quality of 0.6783 were used (Supplementary Fig. 6). The simulation was executed on a local processing computer with 16 computational cores.

### 5.11 Invasion assays

The Lumina-chip was used for cancer cell invasion studies in four conditions (Supplementary Fig. 2); *(1) Cancer cell invasion without vessel (*Supplementary Fig. 2A*).* After collagen I (2.5 mg/ml) was introduced in the middle channel and polymerized, the MCF-7 or MDA-MB-231 tumoroids were suspended in Matrigel-supplemented RPMI media (10 % v/v). Next, 10 μl of the suspension was introduced into the ductal lumen. The Lumina-Chip was kept in culture for 8 days, and the medium was refreshed every day; the medium was refreshed only in the other lumen; the old media was aspirated from both ductal lumen reservoirs and 200 μl of media was added in one of the reservoirs. When the media flowed to the other reservoir due to hydrostatic pressure, another 200 μl of media was added to the same reservoir. The media was composed either of a 1:1 mix of HUVEC and cancer cell media, or of this mix supplemented with 20 % FBS. *(2) Cancer cell invasion in co-culture with vessel* (Supplementary Fig. 2B)*.* When the HUVECs fully covered the vessel lumen after 3 days in culture, the MCF-7 or MDA-MB-231 tumoroids were introduced into the ductal lumen, and the media was changed every day, as explained in condition 1. The system was kept in culture for 8 more days (11 days in total). *(3) Cancer cell invasion from breast epithelial duct* (Supplementary Fig. 2C)*.* MCF10a cells were introduced in the ductal lumen and the chip was kept in culture for 2 days. When the lumens were fully lined with ECs and epithelial cells, the tumoroids were introduced into the ductal lumen and the media was refreshed daily, as explained in condition 1. The system was kept in culture for 8 more days in culture (10 days in total). The media was composed of a 1:1 mix of cancer cells, and MCF10a media. *(4) Cancer cell invasion in triculture with vessel and breast duct* (Supplementary Fig. 2D). HUVECs were cultured for 3 days in the vessel lumen, and MCF10a cells were introduced on the second day in the ductal lumen and the chip was kept in culture for 2 more days (in total 3 days). When the lumens were fully lined with ECs and epithelial cells, the tumoroids were introduced into the ductal lumen and the media was refreshed daily, as explained in condition 1. The system was kept in culture for 8 more days in culture (11 days in total). The media was composed of a 1:1:1 mix of HUVECs, cancer cells, and MCF10a media. The graphs in Fig. 5, Fig. 6 are based on data from at least three regions of interest per chip, and three chips per condition. To compare the number of single MDA-MB-231 cells invading, we assumed an equal initial number of cells in the ductal channel by introducing equal concentrations of MDA-MB-231 tumoroids in the channel. The invasion distance of cancer cells, and the length of the epithelial protrusions were measured with Fiji.

### 5.12 Immunostaining, live imaging and histology

The cell samples were fixed using 1.85 % v/v formaldehyde (Merck, 1040031000) in PBS (Westburg, LO BE02-017F) for 15 min. The samples were permeabilized by exposing them to 0.5 % v/v Triton X-100 (Merck, 108603) solution in PBS for 15 min. Samples were blocked via exposure to blocking solution (1 % w/v Bovine Serum Albumin) (BSA) (Sigma-Aldrich, 9048-46-8), and incubated at room temperature for 1 h. The cell nuclei were stained using NucBlue Fixed Cell ReadyProbe Reagent (Thermo Fisher, R37606) and the cytoskeleton was stained for F-actin using phalloidin (ActinGreen 488 ReadyProbes Reagent, Thermo Fisher, R37110). Staining was performed by exposing the samples to PBS containing 2 drops/mL of each reagent for 60 min. Primary antibodies: goat-anti human VE-Cadherin (AF938, R&D Systems), mouse-anti CD31 (Agilent, M0823), mouse anti-E-Cadherin (Fisherscientific, 10383223). Secondary antibodies: donkey anti-Goat (ThermoFisher Scientific, A32849), goat anti-Mouse (ThermoFisher Scientific, A28180). Before and after each of the previous steps, the sample was washed three times in PBS. Pure collagen samples were not fixed, but labeled directly using a CNA35-OG probe, as previously described [74]. The live imaging assays were recorded using inside incubator microscopes (Axion biosystems-Lux). Fixed samples were imaged by a Thunder-imaging system (Leica-microsystems) fluorescent microscope.

### 5.13 Statistical analysis

Statistical analysis was performed using one-way ANOVA with Tukey's multiple comparisons test in GraphPad Prism 9 software (GraphPad Software Inc., San Diego, CA, USA). Differences were considered to be significant when p < 0.05.

## CRediT authorship contribution statement

**Mohammad Jouybar:** Writing – review & editing, Writing – original draft, Visualization, Validation, Software, Methodology, Formal analysis, Data curation, Conceptualization. **Oscar Stassen:** Writing – review & editing, Methodology. **Hamed Moradi:** Writing – original draft, Visualization, Validation, Software, Methodology, Formal analysis. **Pan Zuo:** Methodology, Formal analysis. **Jaap M.J. den Toonder:** Writing – review & editing, Supervision, Resources, Project administration, Funding acquisition.

## Funding

This work was supported by the Institute of Complex Molecular Systems (ICMS), the European project Moore4Medical [10028031], and by the 10.13039/501100003246Dutch Research Council 10.13039/501100003246NWO (grant number Science-XL 2019.022, ‘The Active Matter Physics of Collective Metastasis'). Moore4Medical has received funding within the 10.13039/501100011688Electronic Components and Systems for European Leadership Joint Undertaking (10.13039/501100011688ECSEL JU) in collaboration with the European Union's H2020 Framework Programme (H2020/2014–2020) and National Authorities, under grant agreement H2020-ECSEL-2019-IA-876190 www.moore4medical.eu. The authors declare no competing financial interests.

## Declaration of competing interest

The authors declare that they have no known competing financial interests or personal relationships that could have appeared to influence the work reported in this paper.

## Figures and Tables

**figs1:**
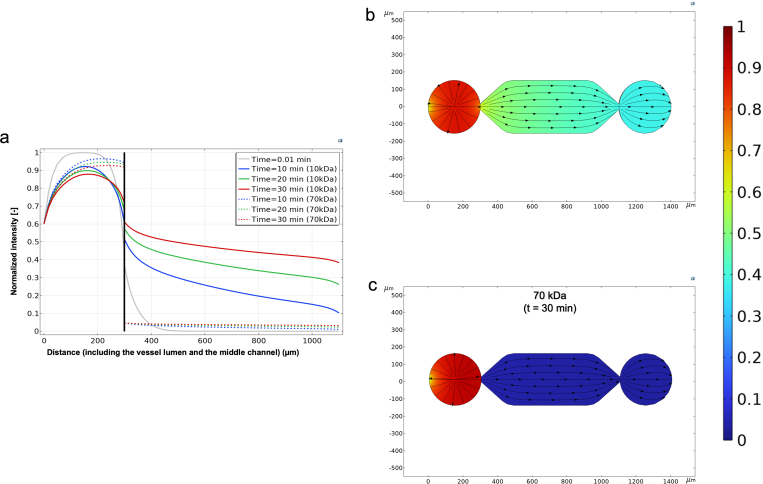


**figs2:**
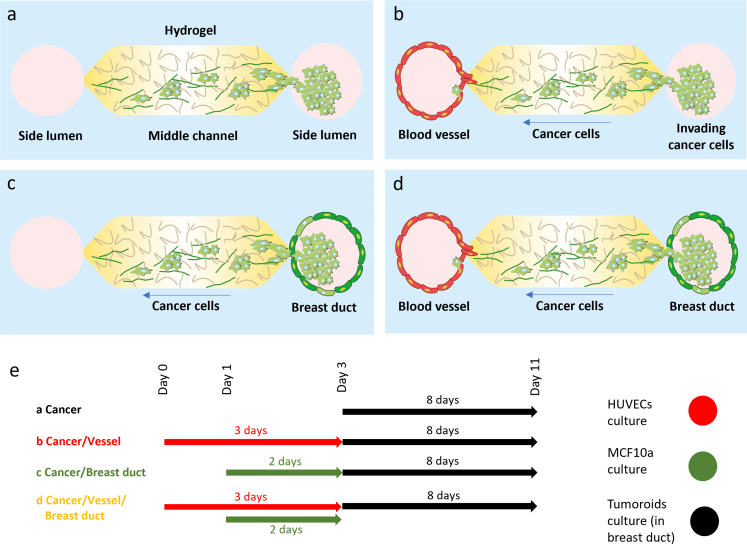


**figs3:**
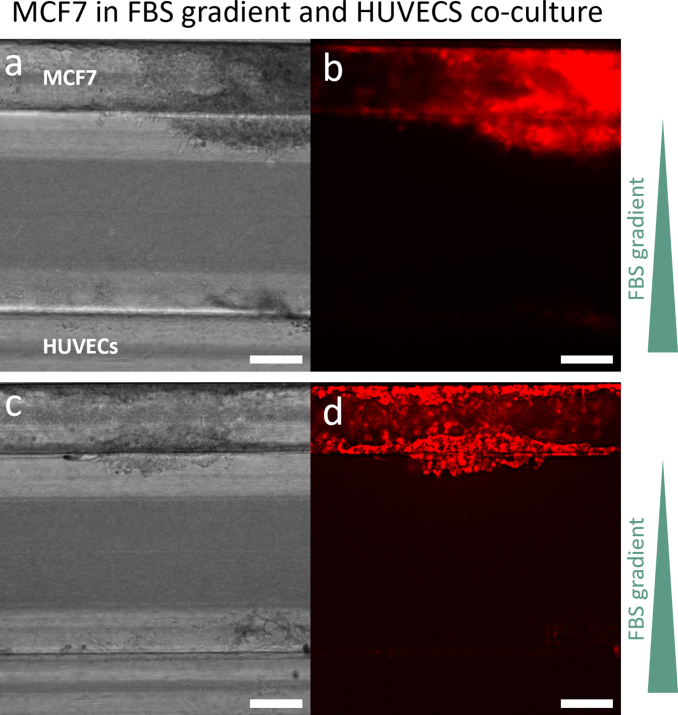


**figs4:**
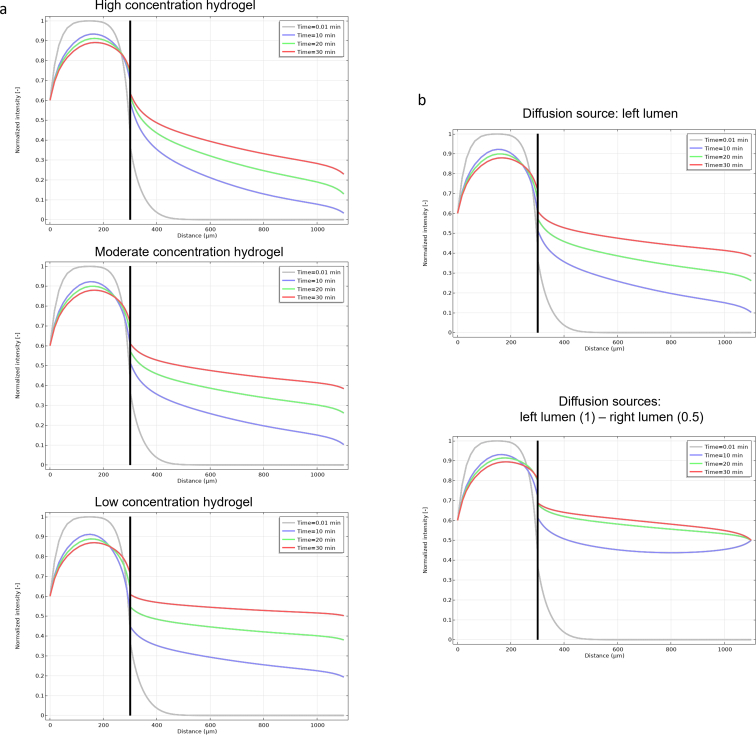


**figs5:**
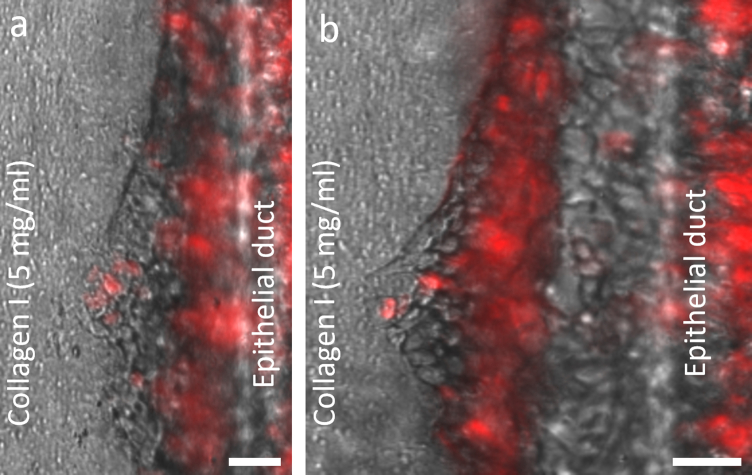


**figs6:**
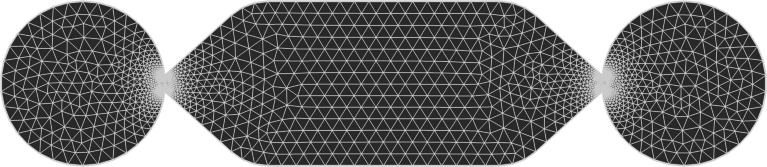


## Data Availability

Data will be made available on request.
