# Supplementary material for: Leveraging femtosecond laser machining for the fabrication of tubular-based Organ-on-Chip systems: modeling cancer metastasis from invasion to intravasation
Source: Mater Today Bio. 2025 May 29;33:101926. doi: 10.1016/j.mtbio.2025.101926 (PMC12205662; doi:10.1016/j.mtbio.2025.101926)
Supplement: Multimedia component 1 [file mmc1.pdf]

Supplemental information

Leveraging femtosecond laser machining for the fabrication of tubular-based Organ-on-Chip systems: modeling cancer metastasis from invasion to intravasation

Mohammad Jouybar, Oscar Stassen, Hamed Moradi, Pan Zuo, and Jaap M.J. den Toonder*

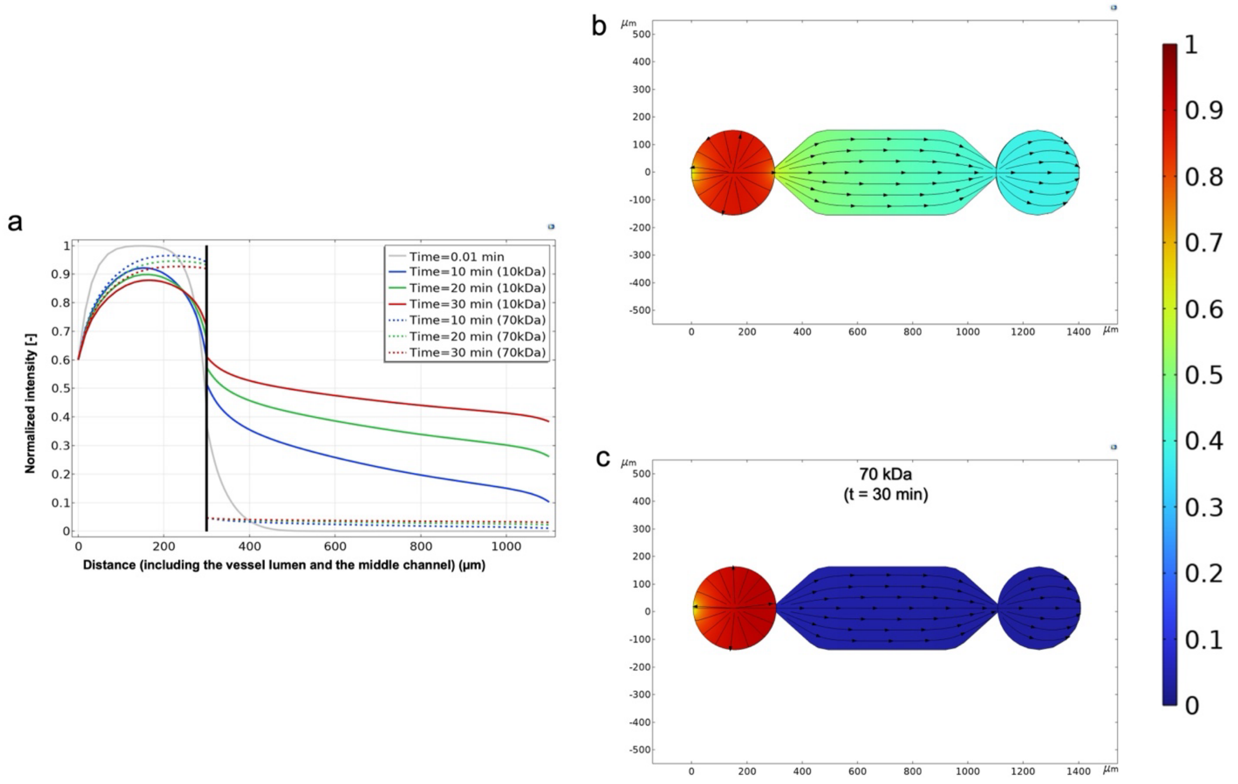

Supplementary Figure 1. Numerical model of permeability assays in the Lumina-Chip. To model the permeability dynamics of the vessel over time, we carried out a numerical simulation of the dextran assay (experimental results shown in **Figure 3**). **a**, The normalized intensity of the molecules was dependent on the permeability coefficient used as a boundary condition at the interface between the vessel and the matrix region in the middle channel. For lower values of the permeability coefficient, the normalized dextran concentration profiles indicate an enhancement in the matrix region as expected. In contrast, for higher values, dynamic diffusion into the middle channel is minimal within the first 30 minutes. **b**,**c**, Diffusive permeability contours obtained from the numerical simulations for (**b**) high and (**c**) low permeability, analogous to smaller molecules (e.g., 10 kDa) and larger molecules (e.g., 70 kDa), respectively.

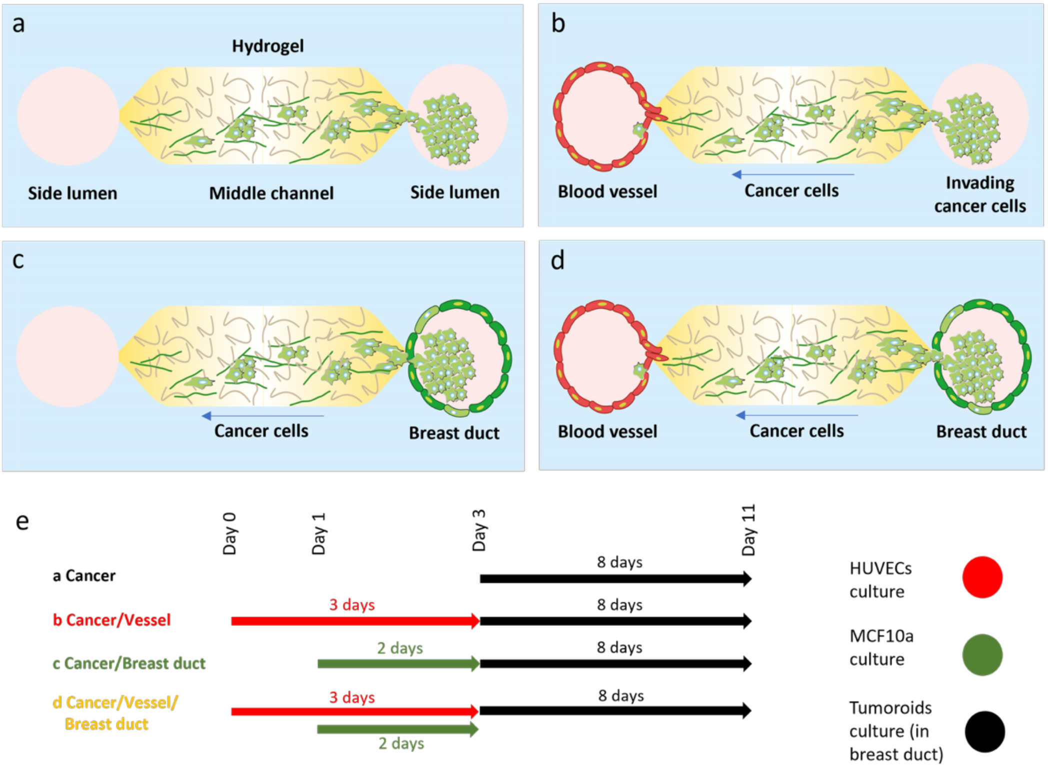

Supplementary Figure 2. Invasion experiment conditions and their corresponding time window. a-d, Different culture conditions for invasion experiments, including, **a**, Tumoroid invasion, **b**, Tumoroid invasion in co-culture with a vessel, **c**, Tumoroid invasion from an epithelial duct, and **d**, Tumoroid invasion from an epithelial duct in co-culture with a vessel. **e**, Time windows for different culture conditions in the invasion experiments.

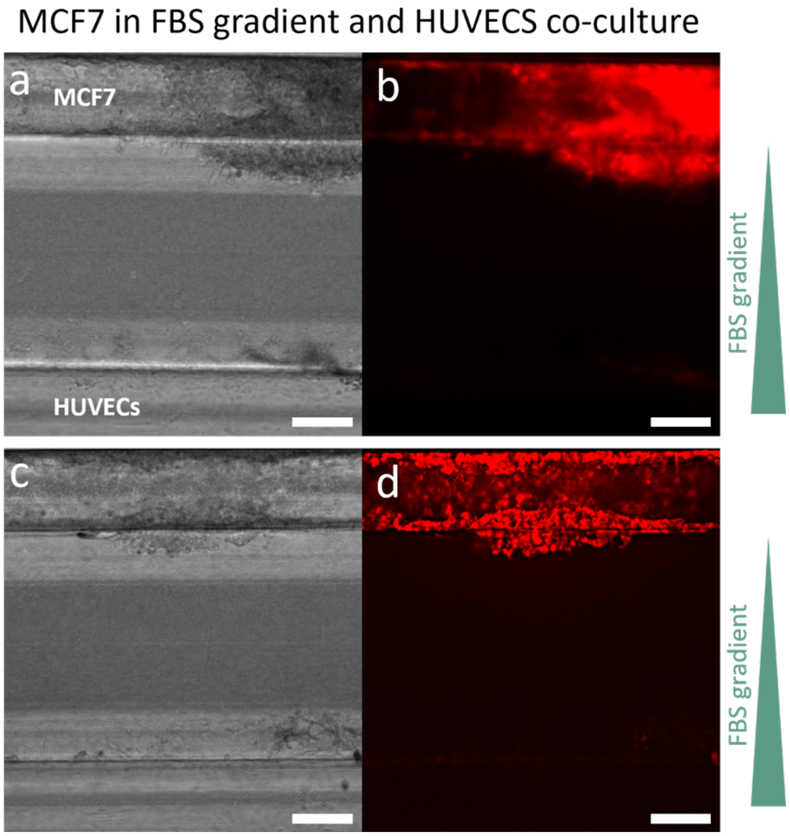

Supplementary Figure 3. MCF-7 tumoroid behavior in the ductal lumen, in co-culture with HUVECs and under an FBS gradient. Phase contrast and fluorescent images represent MCF-7 cells (red) in the ductal lumen, slightly pushing into the collagen I after filling in the channel (DCIS), while HUVECs line the other lumen. **a-b** and **c-d** show examples of regions of interest in two chips. Scale bars, 200 µm.

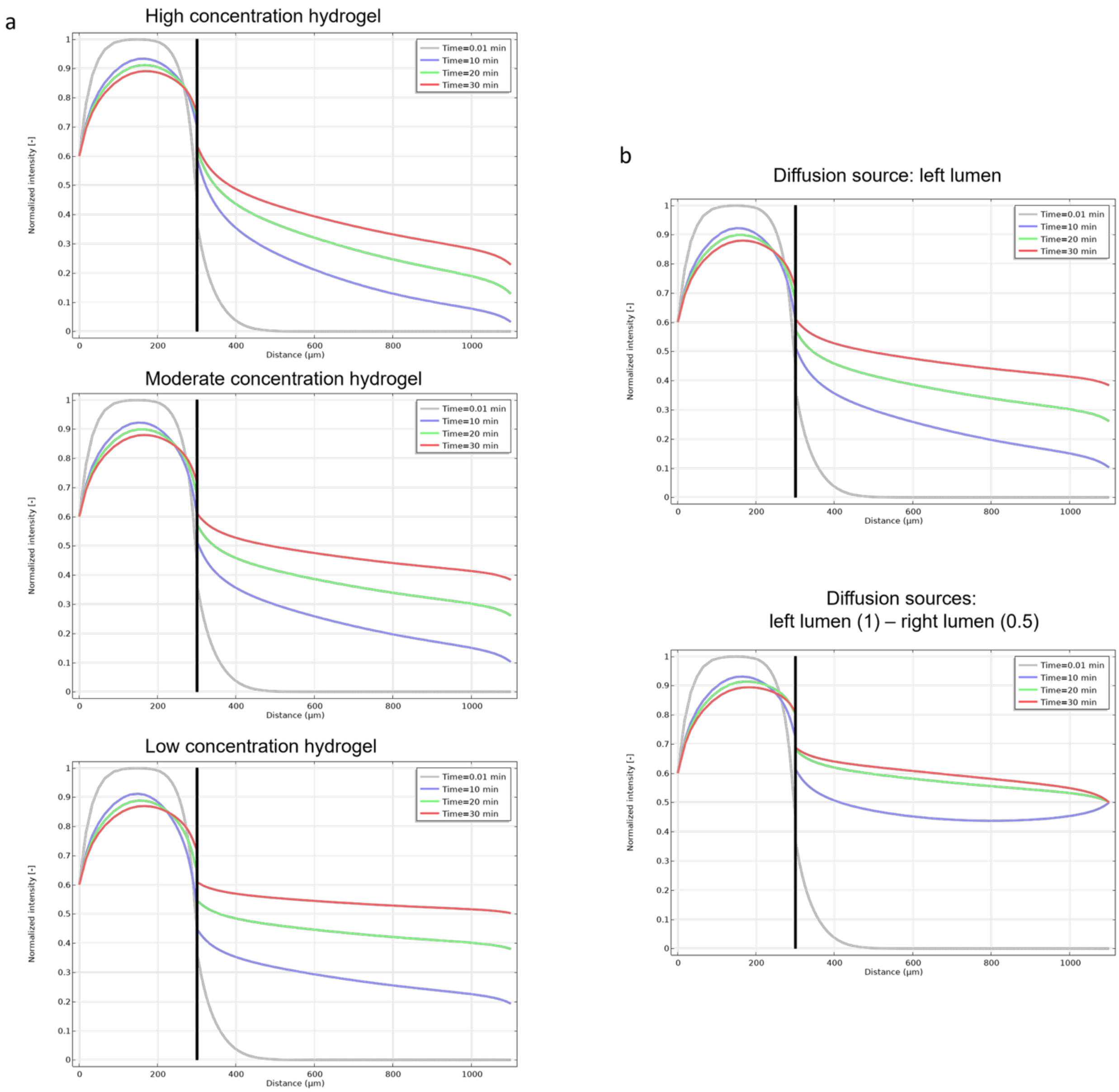

Supplementary Figure 4. Numerical models for predicting different experimental scenarios. a, Diffusive profiles showing the normalized dextran concentration in time for high, moderate and low concentration hydrogels correlated with different diffusive permeability. **b**, Diffusive profiles showing the normalized dextran concentration in time with one dextran source (left: 1), and two dextran sources (left: 1, right: 0.5).

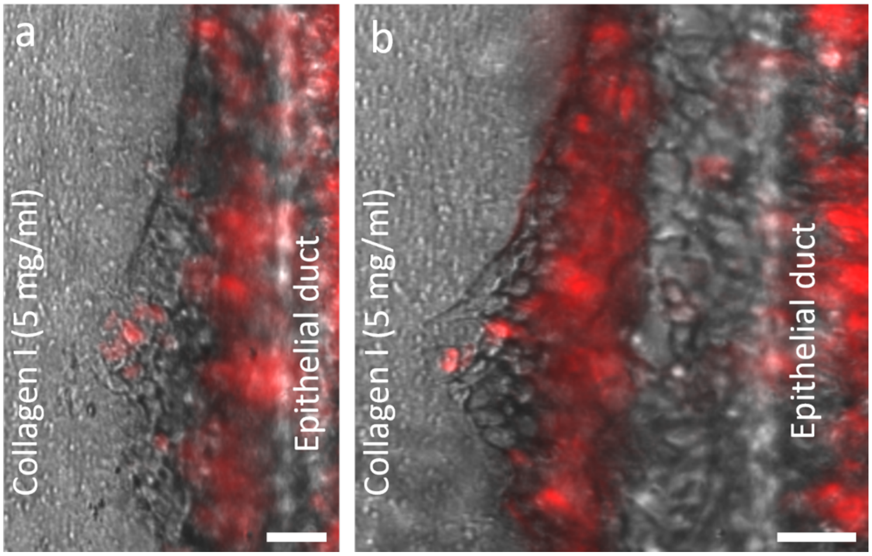

Supplementary Figure 5. MDA-MB-231 cancer cells (red) were often located at the tip of epithelial protrusion when the high concentration collagen I (5 mg/ml) was used. Scale bars, 30 µm.

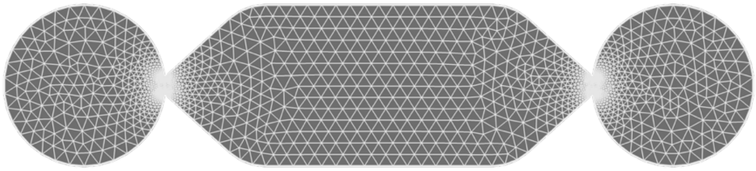

Supplementary Figure 6. Mesh configuration for the cross-section of the Lumina-Chip. A total of 250k unstructured tetrahedral grids were generated in the computational domain.

Supplementary Video 1. Time-lapse video of cancer cell intravasation. A single MDA-MB-231 cell, after invasion from an epithelial protrusion, intravasates into the vessel. Two HUVECs open the way for the traverse of the cancer cell.
